# The relationship of endotoxaemia to peripheral and central nervous system inflammatory responses in Human African Trypanosomiasis

**DOI:** 10.1017/S0031182016002158

**Published:** 2016-11-29

**Authors:** LORNA MACLEAN, ELTAYB A. ABOUBAKER, PETER G. E. KENNEDY, JEREMY M. STERNBERG

**Affiliations:** 1 Drug Discovery Unit, Division of Biological Chemistry and Drug Discovery, James Black Centre, College of Life Sciences, University of Dundee, Dundee DD1 5EH, UK; 2 Institute of Biological and Environmental Sciences, University of Aberdeen, Zoology Building, Aberdeen AB24 2TZ, UK; 3 Institute of Infection, Immunity and Inflammation, College of Medical, Veterinary and Life Sciences, University of Glasgow, Glasgow G61 1QH, UK

**Keywords:** Human African trypanosomiasis, *Trypanosoma brucei rhodesiense*, endotoxaemia, inflammatory response, cerebrospinal fluid

## Abstract

Endotoxaemia has been described in cases of Human African trypanosomiasis (HAT), but it is unclear if this phenomenon influences inflammatory pathology either in the periphery or central nervous system (CNS). We studied endotoxin concentrations in the plasma and cerebrospinal fluid (CSF) of *Trypanosoma brucei rhodesiense* patients using the chromogenic *Limulus* Amoebocyte lysate assay. The relationship of endotoxin concentration to the presentation of gross signs of inflammation and the inflammatory/counter-inflammatory cytokine profile of the relevant compartments were analysed. We demonstrate that HAT patients exhibit parasitaemia-independent plasma endotoxaemia, and that this is associated with splenomegaly and lymphadenopathy. Endotoxin concentrations normalize rapidly after treatment. There was no evidence of endotoxin release in the CNS. A rapid normalization of endotoxin levels after treatment and lack of association with parasitaemia suggest that gut leakage is the main source of endotoxin in the circulation. Low CSF endotoxin concentrations and a lack of any association with neuroinflammatory markers or neurological sequelae suggest that endotoxin does not play a role in the pathogenesis of the disease in the CNS.

## INTRODUCTION

Human African trypanosomaisis (HAT), also known as sleeping sickness, is caused by infections with the tsetse fly vectored haemoflagellates *Trypanosoma brucei gambiense* (West Africa) and *T. b. rhodesiense* (East Africa). In both cases, infection progresses from an initial haemolymphatic (early stage) to a meningoencephalitic (late stage) infection in which parasites penetrate the central nervous system (CNS), resulting in neuro-inflammatory disease and ultimately death if untreated (Kennedy, 2013). Dysregulation of inflammatory responses and in particular overactivation of type 1 macrophage responses is associated with pathology in both clinical disease and experimental animal infection (Sternberg and Maclean, 2010). This phenomenon is driven by MyD88-dependent pathways (Drennan *et al.* 2005). In the CNS, toll-like receptor (TLR)2 and TLR9, but not TLR4 ligands have been implicated in mediating inflammatory responses (Amin *et al.* 2012), but it is not known whether this is also the case systemically. Endotoxins are potent inflammatory activators signalling in myeloid and other cells via an interaction between the lipid A component and TLR4 receptors (Raetz and Whitfield, 2002). Because of baseline levels of gastrointestinal tract permeability, low levels of endotoxin are found in the plasma from healthy subjects; however, in pathological conditions such as severe bacteraemia, these increase massively and may result in endotoxic shock syndrome (Brandtzaeg *et al.* 1989). Studies in experimental animals have demonstrated endotoxaemia in *T. brucei* infection (Pentreath, 1994). The origin of the endotoxins causing this phenomenon is unclear. Evidence has been presented that intestinal permeability of microbial products, which is the basis of baseline levels of serum endotoxin, is involved (Nyakundi *et al.* 2002) and is consistent with the lack of correlation of endotoxaemia to parasitaemia. However, contradictory reports indicate a non-gut origin and an association with parasite membrane components (Ngure *et al.* 2009*a*, *b*).

A key question in establishing the significance of endotoxaemia in African trypanosomiasis is to determine if it is observed in clinical cases and if so whether there is a relationship to pathogenesis. In a previous study, data were presented on endotoxaemia in *T. b. gambiense* HAT patients. Here plasma and CSF endotoxin concentrations were increased during infection compared with non-infected individuals (Pentreath *et al.* 1996), and the *Limulus* Assay results were independently confirmed by measurement of antibody responses to endotoxin (Pentreath *et al.* 1997). However, these studies did not include data that would allow analysis of the relationship of endotoxin levels to disease progression, in terms of parasitaemia, diagnostic stage or development of the inflammatory response.

As part of a larger study of the role of inflammatory cytokines in the pathogenesis of *T. b. rhodesiense* HAT, we have analysed a set of plasma and cerebrospinal fluid (CSF) samples from patients and sympatric controls in Eastern Uganda using the *Limulus* amoebocyte lysate (LAL) assay (Hurley, 1995). For each sample, detailed information on stage of infection, clinical signs and inflammatory responses (plasma and CSF cytokines and white cell counts) were recorded. These enabled us to test the hypothesis that endotoxaemia occurs in *T. b. rhodesiense* HAT and investigate the relationship of plasma and CSF endotoxin levels to CNS and systemic inflammatory responses.

## MATERIALS AND METHODS

### Study subjects

This study was conducted according to the principles expressed in the Declaration of Helsinki. All patients recruited received written and verbal information explaining the purpose of this study and gave informed written consent. Where participants were minors, consent was also given by the parent or legal guardian.

All protocols were approved by ethics committees in Uganda (Ministry of Health Ref ADM130/313/05) and UK (Grampian Joint Ethics Committee Ref 02/0061).

69 Patients and 18 controls were recruited at the LIRI Hospital (Tororo District, Uganda) and Serere Health Centre (Serere District, Uganda) between August 2002 and July 2003, part of a larger multicentre study for which all protocols (disease staging, clinical and neurological parameters) have been described elsewhere (MacLean *et al.* 2010). The selection of patient samples for this study was entirely random and driven by limitations in available sample volume for the *Limulus* assay. Patient demographic and clinical data are summarized in Table 1. All plasma and CSF samples were collected aseptically. Blood was collected in EDTA vacutainers (Vacuette, Greiner Bio-one Ltd., UK) and centrifuged at 10 000 rpm for 10 min. The 4 mL of plasma was aliquoted into 2 mL cryovials (Greiner Bio-one Ltd., UK) and frozen immediately in liquid nitrogen. CSF was collected in 15 mL sterile polypropylene tubes (Greiner Bio-one Ltd., UK) and centrifuged for 10 min at 10 000 rpm. CSF supernatant was aliquoted into 2 ml cryovials frozen immediately in liquid nitrogen. All plasticware was certified endotoxin free.

**Table 1.** Study population and disease characteristics

Population	Infected *n* = 69	Control *n* = 18
Gender % female	57%	55%
Age (median, IQR)	20 (11–35)	37·5 (28–50)
Packed cell volume (%, IQR)	29 (25–34)	40 (36–42)
Late stage % parasitaemia (median, range)^a^	49/69 2 (0–100)	
Reported duration, days (median, IQR)	35 (11–35)	
Lymphadenopathy	30%	
Splenomegaly	17%	
Hepatomegaly	3%	
Tremor	54%	
Ataxia	59%	
Somnolennce	49%	
Cranioneuropathy	19%	
GCS<15	19%	

a Parasitaemia estimated from parasites observed in 10 micropscope fields at × 400 on thick films as described in(MacLean *et al*., 2010).

Samples were maintained in liquid nitrogen (including vapour phase air freight) and subsequently transferred to −80 °C until required for analysis.

### CSF and plasma endotoxin measurement

Plasma and CSF samples were diluted with PBS (phosphate-buffered saline) 1:5 and heated at 70 °C for 10 min. After centrifugation (13 000 g) for 5 min, the supernatant was decanted and endotoxin activity was measured using a chromogenic LAL assay (QCL-1000, Lonza, UK) according to the manufacturer's protocol. Every assay included a series of standards from 0·05–2 EU mL^−1^ calibrated using a standardized lot of *Escherichia coli* 011:B4 endotoxin (Lonza E50–640, UK) and the biological limit of detection were calculated for each assay. Limits of detection were in the range 0·1–0·05 EU mL^−1^. The effectiveness of heat treatment was confirmed by inclusion of a control sample of plasma spiked with a 0·2 EU mL^−1^ final concentration of endotoxin standard. Because previous literature on endotoxaemia in HAT have presented endotoxin concentrations as lipopolysaccharide (LPS) equivalents, endotoxin concentrations were expressed as pg mL^−1^ LPS using the conversion 1 EU mL^−1^ = 100 pg mL^−1^ (Hurley, 2013).

### Plasma and CSF cytokine assays

Interferon (IFN)-γ, interleukin (IL)-6, transforming growth factor (TGF)-*β* and IL-10 concentrations were measured using a solid-phase sandwich ELISA (OptiEIA; BD Pharmingen, Oxford, UK), as described previously (MacLean *et al.* 2007). Biological limits of detection were 1·8, 8·3, 19·2 and 1·6 pg mL^−1^, respectively

### Statistical analysis

Endotoxin concentration and cytokine data were right skewed and multimodal. Therefore non-parametric inferential statistical analyses were used (tests as indicated in figure legends or text) and were carried out using JMP10·0 (SAS Institute, Cary, NC, USA). Biological limits of detection were calculated as the mean blank value plus 2 s.d. plus 2 s.d. of the lowest standard. For descriptive and inferential statistical analysis, results below the limit of detection were assumed to be (0·5 × limit of detection value).

## RESULTS

### Endotoxaemia in patients and controls

**Fig. 1.**
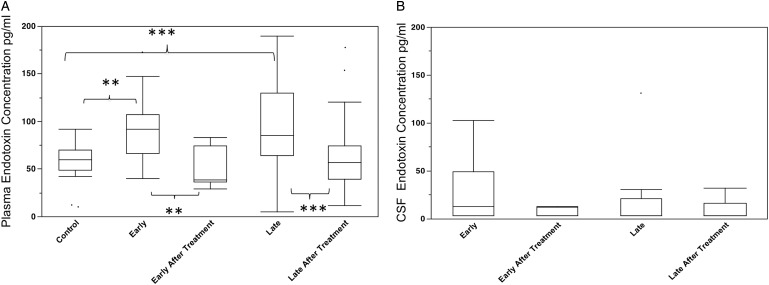
Endotoxaemia in control (*n* = 18), early stage (*n* = 20) and late stage (*n* = 49) HAT patients on admission and after treatment for sleeping sickness. (A) Plasma endotoxin concentration (B) CSF endotoxin concentration. Box (median, quartiles), whiskers (10–90th percentile) and outliers. **P < 0·01;***P < 0·001 Kruskal–Wallis test with Dunn's multiple comparison adjusted post-test.

Endotoxin concentration was measured using a chromogenic end-point LAL assay using heat-treated samples to inactivate inhibitory factors as described in (Hurley, 1995). The elimination of inhibitors was confirmed by the inclusion of a control sample spiked with LPS (0·2 EU mL^−1^) in each assay. A recovery of ⩾80% activity was measured provided samples were treated at 70 °C for at least 10 min. Plasma endotoxin concentration was significantly increased in HAT patient samples compared with control subjects. There was no significant difference between early (haemolymphatic) and late (meningoencephalitic) stage HAT cases, and in all patients plasma endotoxin levels reduced to control levels after chemotherapeutic treatment for HAT (Fig. 1A). The overall median endotoxin level in HAT patients was 91 pg mL^−1^ (IQR 67–117 pg mL^−1^). There was no significant effect of subject gender on overall plasma endotoxin concentration and significant endotoxaemia was evident in both male and female HAT patients (Table 2). Also there was no significant relationship between subject age and plasma endotoxaemia within the HAT patients group [Spearman's rank correlation coefficient (*ρ*) = −0.14 *P* = 0·57; not significant (NS)].

**Table 2.** Endotoxaemia in HAT patients by disease stage and gender

	Plasma endotoxin concentration pg mL^−1^ (median, IQR)	
	Male (*n* = 38)	Female (*n* = 49)
Control (*n* = 18)	60·1 (19·5–68·0)	59·7 (56·0–72·5)
Early (*n* = 20)	88·0 (67·25–115·0)*	93·5 (64·4–107·8)*
Late (*n* = 49)	107·2 (61·1–142·5)**	77·5 (64·0–112·2)*

*Significantly increased over control *P* < 0·05 Dunn's *post hoc* test.

**Significantly increased over control *P* < 0·01 Dunn's *post hoc* test.

Within the HAT patient group (both early and late stages) there was no apparent relationship between the plasma endotoxin level and parasitaemia (Spearman *ρ* = 0·03; NS), reported duration of infection (*ρ* = 0·08; NS) or anaemia as measured by packed cell volume (*ρ* = −0·014; NS).

### CSF endotoxin concentrations

For ethical reasons we did not obtain CSF from control individuals. CSF from patients was assessed for pleiocytosis, which ranged from 1 to 400 cells *µ*L^−1^ and parasitosis, which ranged from 0 to 80 trypanosomes *µ*L^−1^. The criteria for a late-stage diagnosis were > 5 white cells *µ*L^−1^ and/or presence of parasites (MacLean *et al.* 2010). In most patient CSF samples, endotoxin levels were low (medians at the limit of detection 5 pg mL^−1^) with outliers exhibiting endotoxin concentrations up to 102 pg mL^−1^ in the early stage and 131 pg mL^−1^ in the late stage (Fig. 1B). There was no relationship between CSF endotoxin concentration and CSF parasitosis (*ρ* = 0·3; NS) or CSF pleiocytosis (*ρ* = 0·31; NS). All HAT patients underwent a second CSF collection after drug therapy, and analysis of these samples revealed no significant difference between pre- and post-treatment CSF endotoxin concentrations.

There was a weak but significant relationship between plasma endotoxin and CSF endotoxin concentration in patients (*ρ* = 0·51 *P* < 0·02), but this was not evident in post-treatment cases (*ρ* = −0·25; NS).

### Relationship of gross inflammatory and cytokine responses to endotoxaemia

Gross inflammatory disease was assessed by physical examination on admission. No relationships were observed between plasma endotoxaemia and either hepatomegaly or pyrexia. However, the occurrence of splenomegaly and cervical lymphadenopathy were significantly associated with plasma endotoxin concentration (*P* < 0·05, logistic regression, likelihood-ratio test). The odds ratios (OR [95% CI]) for the occurrence of splenomegaly and cervical lymphadenopathy were 1·19 [1·01–1·4] and 1·16 [1·02–1·35] respectively for every 10 pg mL^−1^ increase in plasma endotoxin concentration. Plasma IFN-*γ*, IL-6, IL-10 and TGF-*β* concentrations were significantly increased in patients compared with controls (Table 3), and of these IFN-*γ* and TGF-*β* concentrations were significantly associated with plasma endotoxin concentration. However, this association was confounded by the increase in plasma endotoxin in HAT cases, and when correlation analysis was carried out within the patient group only all associations became non-significant.

**Table 3.** Plasma and CSF cytokines in infected and control groups and relationship to endotoxaemia

	Plasma cytokine concentration (pg mL^−1^) median (IQR)			CSF cytokine concentration (pg mL^−1^) Median (IQR)		
Analyte	Control (*n* = 18)	Infected (*n* = 69)	Relationship to plasma endotoxin (Spearman *ρ*)	Early stage (*n* = 20)	Late stage (*n* = 49)	Relationship to CSF endotoxin concentration (Spearman *ρ*)
IFN-*γ*	37·5 (26·6–48·2)	75·5 (31·9–124·6)*	0·24 *P* < 0·05^a^	12·5 (6·8–28·5)	19·2 (8·6–48·5)	−0·03 NS
IL-10	17·9 (6·2–33)	93·2 (54·2–163·8)***	0·12 NS	8·9 (5·9–11·6)	104·3 (34·0–240·2)***	−0·24 NS
TGF-*β*	25·5 (19·7–27·6)	1014 (394·8–3680)***	0·31 *P* < 0·01^b^	210·3 (112·7–346·1)	100·6 (70·6–133·6)***	0·22 NS
IL-6	BLD	38·5 (22·8–86·6)***	0·28 NS	14·2 (7·1–20·7)	62·6 (5·6–186·2)**	−0·3 NS

*P < 0·05; **P < 0·01; ***P < 0·001 (Dunn's *post hoc* test).

a In HAT group only *ρ*  =  0·19 NS.

b In HAT group only *ρ*  =  0·2 NS.

In the CSF, stage progression was associated with significant increases in CSF IL-6 and IL-10 concentrations and a reduction in CSF TGF-*β* levels (Table 3).

However, there were no significant relationships between CSF cytokine concentration and endotoxin concentration. Patients were also assessed for neurological sequelae of HAT (tremor, ataxia, somnolence) and Glasgow Coma Score and no relationship to CSF endotoxin concentration was detected.

## DISCUSSION

Experimental model studies have demonstrated that both systemic innate responses to African trypanosomes and CNS invasion depend on the activation of type 1 inflammatory responses that are, at least in part, driven by MyD88-dependent signalling (Drennan *et al.* 2005). In experimental models and in the clinic, systemic and CNS inflammatory responses underlie disease pathogenesis (Sternberg and Maclean, 2010). Mediators that may be involved in these processes include components of the GPI anchor of the trypanosome variant surface glycoprotein and CpG dinucleotides. Several studies have also provided evidence of endotoxaemia in experimental models of trypanosomiasis (Pentreath, 1994) and also it has been demonstrated that endotoxin and endotoxin antibody levels are increased in clinical cases of African trypanosomiasis caused by *T. b. gambiense* (Pentreath *et al.* 1996; Pentreath *et al*. 1997). These findings suggested that endotoxins may also act as an immune-modulating factor promoting inflammatory responses and pathology in HAT.

We studied clinical plasma and CSF samples from *T. b. rhodesiense* HAT patients to determine the relationship of endotoxaemia to disease using the LAL assay. Unlike previous studies using plasma and CSF from *T. b. gambiense* patients (Pentreath *et al.* 1996), the detailed parasitological and clinical data associated with the HAT patients in this study enabled us to determine the relationships of endotoxin concentrations to parasitaemia, gross inflammatory responses and cytokine markers of inflammation.

The LAL assay is susceptible to a range of interfering factors in blood, serum and plasma (Gnauck *et al.* 2016) including LPS-binding proteins. These were controlled for using dilution and heat treatment, with confirmation from plasma spiked with known concentrations of LPS. Contamination from exogenous LPS, absorption and degradation of samples was controlled by the collection of all samples in identical, endotoxin-free vessels, these being then processed contemporaneously and identically, and stored for similar periods.

In *T. b. rhodesiense* HAT patients, plasma endotoxin concentrations were increased compared with control individuals. The range of endotoxin concentration was similar to that reported in *T. b. gambiense* infection (Pentreath *et al.* 1996), and was modest compared with endotoxaemia levels observed in conditions such as septic shock (Brandtzaeg *et al.* 1989). Endotoxin concentrations returned to control levels after drug treatment for trypanosomiasis, confirming the relationship with the infection. We demonstrated that plasma endotoxin concentration was unrelated to parasitaemia, consistent with observations in mouse model infections (Pentreath, 1994) suggesting that a parasite-derived endotoxin-like substance is unlikely to be the cause of endotoxin activity. This conclusion is further supported by the low concentration of endotoxin in CSF even where high levels of parasitosis occurred. Given that endotoxaemia returned to control levels after drug therapy for HAT, it seems unlikely that systemic bacteraemia is involved, and that the most likely basis of the phenomenon is as a result of translocation of microbial products from the gut by either paracellular permeability or transcellular passage (Gnauck *et al.* 2016). This would be consistent with the enhanced gut translocation of non-metabolizable sugar probes observed in a rat model of HAT (Nyakundi *et al.* 2002).

As has been previously found in both *T. b. rhodesiense* (MacLean *et al.* 2007) and *T. b. gambiense* HAT (Lejon *et al.* 2002), plasma cytokine markers of both the inflammatory (IFN-*γ*, IL-6) and counter-inflammatory immune response (IL-10, TGF-*β*) were upregulated in patients in this study. While plasma endotoxin concentration was not related to the concentrations of either IL-6 or IL-10, there were significant correlations to IFN-*γ* and TGF-*β* plasma concentrations. This suggests a complex interplay between endotoxaemia and the cellular inflammatory response. Endotoxaemia was significantly associated with the occurrence of two gross inflammatory manifestations of infection, namely splenomegaly and lymphadenopathy.

In the CSF, endotoxin concentrations were low and were unrelated to stage progression, or neuroinflammatory responses as measured by pleiocytosis and inflammatory cytokine expression. The increased concentrations of IL-6, IL-10 and decreased concentration of TGF-*β* in late-stage patients is consistent with previous findings (MacLean *et al.* 2007), but was unrelated to endotoxin concentration. There was a weak relationship between plasma and CSF endotoxin concentration suggesting that cases with significant CSF endotoxin levels could reflect blood brain barrier penetration, but the lack of any association to neuro-inflammation and neurological sequelae associated with CNS infection by trypanosomes indicates that endotoxins play a minimal role in late-stage disease. Our results are not consistent with those obtained in *T. b. gambiense* HAT (Pentreath *et al.* 1996) where mean CSF endotoxin concentrations of 45–50 pg mL^−1^ were observed, with a strong correlation to plasma endotoxaemia. This could reflect differences in pathobiology between the two subspecies of parasite, but importantly the previously reported *T. b. gambiense* data did not distinguish early and late-stage cases and thus precluded any analysis of the relationship between CSF endotoxin and the presence of parasites in the CNS.

In conclusion, we demonstrate that *T. b. rhodesiense* HAT is associated with low-grade systemic endotoxaemia. We reasoned that the source of endotoxin in this disease is almost certainly parasite-independent, and most probably is a result of permeability of the gastrointestinal tract. Such an increase in permeability might be induced by the inflammatory response itself (Hietbrink *et al.* 2009), and this possibility is amenable to testing in inflammatory signalling defective mouse models such as *MyD88*^−/−^ (Drennan *et al.* 2005). Endotoxaemia may play a role in the peripheral inflammatory pathology of HAT but appears to be unrelated to late-stage neuroinflammatory disease.
